# Merkel cell stimulation in fear and sensory signaling

**DOI:** 10.1038/s41386-025-02144-w

**Published:** 2025-06-07

**Authors:** Austin C. Korgan, Rodrigo Orso, Isabelle J. Sibley, Kathryn E. Prendergast, Tanja Jovanovic, Tracy L. Bale

**Affiliations:** 1 Department of Psychiatry, University of Colorado Anschutz Medical Campus, Aurora, CO USA; 2 Department of Psychiatry and Behavioral Neurosciences, Wayne State University, Detroit, MI USA

**Keywords:** Stress and resilience, Cellular neuroscience

## Abstract

Stress and traumatic experiences have significant and lasting effects on sensory systems. We recently identified unique expression of proteins associated with epidermal skin cells (keratinocytes) and mechanosensory Merkel cells (MC) in circulating extracellular vesicles from adult women who had experienced sexual trauma specifically during adolescence, biologically linking trauma exposure with a specific neuron-like skin cell. Here, we aimed to develop and validate a preclinical mouse model utilizing chemogenetic (DREADD Gq) activation of a population of MC. Using a reporter line, we confirmed the expected pattern of the Krt14 Cre in specific MC skin areas and that these tissues expressed relevant MC marker genes similarly between male and female mice. Chemogenetic stimulation of MC produced robust neuronal activation of the insular cortex (IC), a brain region relevant to somatosensory and valence integration. To determine if the mice could detect MC activation, home cage behaviors following CNO treatment significantly increased nest grooming time. Conditioned place preference further revealed an avoidance response following MC stimulation; an effect that was stronger in female mice. Finally, to connect back to our trauma question, we examined MC activation in fear conditioning and identified deficits in fear extinction. Overall, these studies validate utilization of this preclinical model in further investigating the mechanosensory system and its potential involvement in PTSD symptoms and therapeutic interventions. Ongoing studies will focus on critical developmental periods relevant to both MC development and sex differences associated with trauma vulnerability and potential sensory based therapeutic options for PTSD-related symptoms.

## Introduction

The impact of severe trauma on the central nervous system (CNS) has been the focus of post-traumatic stress disorder (PTSD) research and treatments [1]. Changes to the structure and function of brain regions that process threat learning, memory, and symptomatology are effective targets for identifying potential treatment options. However, recent evidence suggests that sensory systems are also susceptible to alterations following severe trauma and may underlie vulnerabilities important for identifying individuals at risk for developing PTSD [2–10]. Similarly, fMRI and fear conditioning data suggest deficits in fear extinction in women who experienced violent trauma [11–16]. Survivors of sexual trauma also show decreased activation of the somatosensory and insular cortices [5, 17, 18]. These studies highlight the importance of identifying the mechanisms by which somatosensory systems are involved in trauma exposure and PTSD risk.

We recently demonstrated that trauma experienced during key developmental periods increased the expression of proteins associated with epidermal skin cells (keratinocytes) and mechanosensory Merkel cells (MC) in circulating extracellular vesicles (EVs) [14]. MC are the only keratinocyte-derived neuron-like mechanosensory cells in the epidermis of skin and are responsible for the detection of light touch in touch domes of hairy skin and rete ridges of glabrous skin [19–21]. MC synapse with Aβ-afferents and express the mechanosensitive ion channel, Piezo2 [22–26]. Piezo2-containing mechanoreceptors and afferents are associated with social and reproductive functions, and disruptions in their function have been implicated in neurodevelopmental disorders including attention-deficit/hyperactivity disorder and autism spectrum disorders [27–31]. Further, genes uniquely expressed in keratinocytes and MC have been implicated in preclinical models of PTSD and genome-wide association studies of individuals with PTSD [32–36]. However, there are insufficient tools with which the mechanisms linking MC to lasting effects of stress or trauma can be investigated. Therefore, based on our previous work, we developed a transgenic mouse model that allows for chemogenetic activation of a population of MC-enriched keratinocytes [14].

Overall, our goal was to validate a novel, preclinical model of MC activation in stress- and PTSD-relevant behaviors providing for future studies to utilize this mouse to ask mechanistic questions about the developmental timing of stress experience on sensory systems in the brain. Therefore, utilizing a chemogenetic (Gq-DREADD) approach to acutely activate MC with clozapine N-oxide (CNO), we first examined in vivo imaging and ex vivo gene expression to confirm the correct and limited expression of our transgenic MC markers in male and female mice [37–39]. Next, we examined c-Fos expression in the insular cortex following CNO activation of MC [40–42]. Then, based on similar somatosensory models, we examined the influence of MC stimulation on tactile sensitivity and naturalistic behaviors to determine if mice could sense MC activation [24, 43–47]. Finally, we tested the valence of, and fear responding to, MC stimulation in a translationally relevant PTSD model [29, 30, 48, 49]. These studies validate the use of this novel preclinical model and establish a foundation for continued investigation of MC in the mechanistic underpinnings of type, timing, and sex differences in PTSD.

## Methods

### Animals

Adult (8-12 weeks; *n* = 114) male and female mice were used for all studies. In vivo imaging studies were conducted with heterozygous KRT14^Cre^ mice (Jax stock #018964; Bar Harbor, ME, USA) bred to homozygous floxed Ai14 mice (Ai14^+^; Jax stock #007914). DREADD studies were conducted with heterozygous KRT14^Cre^ mice (MC; Jax stock #018964) maintained on a C57BL/6 J background bred to heterozygous hM3Dq Designer Receptor Exclusively Activated by Designer Drugs (DREADD) mice (DRD^+^; Jax stock #026220) on a mixed C57BL/6J x 129S1/SvlmJ (Jax stock #002448) background to target a MC-enriched population of keratinocytes with CNO administration. Separate cohorts of mice were used for each behavioral task, except conditioned place preference and home cage behavior were conducted on the same cohort, with home cage observations occurring immediately prior to conditioned place preference (CPP) training and 7 days after CPP testing. Mice were administered chow (Teklad 2920X global soy protein-free extruded; Madison, WI, USA) and water *ad libitum*. Lights were maintained on a 10:14 schedule with lights on at 0600 hr and lights off at 2000 h. All animal experiments were approved by the University of Colorado Anschutz Medical Campus Institutional Animal Care and Use Committee and conducted in accordance with the National Institutes of Health Guide for the Care and Use of Laboratory Animals.

### Clozapine N-oxide administration

CNO (Hello Bio #HB6149; Bristol, UK) was prepared in cookie dough at 0.1 mg CNO/g dough (bacon (Transgenic Dough Diet, Bio-Serv #S3472; Flemington, NJ, USA), Nutella (Ferrero, Alba, IT), sugar cookie (Pillsbury; Minneapolis, MN, USA), or Reese’s Peanut Butter (Pillsbury)) [42]. For all CNO dosing experiments, mice were primed with CNO-free dough for 3 days, followed by 2 days of no dough. Acute CNO was administered at 5 mg/kg unless otherwise noted (Table S1). Previous development of DREADD-Gq models in our lab found that varied flavors and dosages of CNO were necessary to maintain consistent consumption of CNO cookie dough, especially if the cell type being activated by CNO is associated with a negative valence [42].

### In Vivo imaging system (IVIS)

Keratinocyte and MC bioluminescence were measured using an in vivo imaging system (IVIS Spectrum, Perkin Elmer; Springfield, IL, USA) in the Anschutz Medical Campus Small Animal Imaging Shared Resource. Ai14^+^ mice were anesthetized with isoflurane and placed in the IVIS. Ai14 imaging was performed with an excitation/emission wavelength pair of 570/620 nm, maximized for TdTomato signal-to-noise ratio.

### TaqMan qPCR gene expression analysis

Mice were anesthetized with isoflurane, treated with Nair for hair removal, and cervically dislocated. Lips and paws were harvested, immediately snap-frozen in liquid nitrogen, and homogenized as previously described [50]. Total RNA was isolated using Qiagen RNeasy Fibrous Tissue Kit according to manufacturer’s protocol (Qiagen #74704; Germantown, MD, USA). cDNA was transcribed using the Applied Biosystems High-Capacity cDNA reverse transcriptase kit (Thermo Fisher #4368814; Waltham, MA, USA). Quantitative real-time PCR (qRT-PCR) was performed using TaqMan Gene Expression Assays, TaqMan Fast Advanced Master Mix (Thermo Fisher #44445556), and primers for KRT7 (Mm00466676_m1), KRT14 (Mm00516876_m1), Piezo2 (Mm01265861_m1), and Actb (Mm02619580_g1). Samples were run in triplicate with a no-template control for each gene in the same qRT-PCR experiment. Relative quantification of gene expression was calculated with the comparative Ct (∆CT) method using *Actb* as a reference gene.

### FFPE preparation of mouse skin

Following isoflurane anesthesia, mouse lips and paws were carefully dissected. Samples were fixed in 10% neutral buffered formalin (Sigma Aldrich, #HT501128) overnight. Samples were then dehydrated in an ethanol gradient (50%, 50%, 80%, 95%, 95%,100%, 100%, xylene x3, and paraffin wax x4) in a Tissue-Tek VIP (Sakura, Torrance, CA, USA) and embedded with Paraplast Plust (Millipore Sigma, #P3683). 4 µm sections were cut on a Microm microtome (HM310) and mounted on TOMO hydrophilic slides (Matsunami Glass Ind., LTD., #TOM-12). Slides were then processed on an Autostainer Link 48 (Agilent Dako, Santa Clara, CA, USA). Slides were incubated (1 hr at 90̊ °C) with Citrate Buffer (pH 6; Bio SB, #BSB 0021, Santa Barbara, CA, USA), washed with TBS-T, blocked in 10% BSA in PBS (1 hr at RT), and rinsed with TBS-T. Slides were then incubated (1 hr at RT) with primary antibodies for TROMA-I anti-cytokeratin 8 (KRT8; DSHB, #AB_531826; 1:300; Iowa City, IA, USA) and HA (Proteintech, 51064-2-AP; 1:100; Rosemont, IL, USA). Sections were then rinsed with TBS-T and incubated with secondary antibodies (1 hr at RT; Alexa Fluor 488 chicken anti-rat IgG; Thermo Fisher #A-21470 and Alexa Fluor 568 goat anti-rabbit IgG; Thermo Fisher #A-11036; 1:2000), washed in TBS-T, and cover-slipped EverBrite Hardset mounting medium containing DAPI (Biotium #23004; Fremont, CA, USA). Images were acquired at 40x magnification on an Olympus APX100 microscope system.

### Immunohistochemistry

Two hours after CNO treatment, mice were anesthetized with isoflurane and transcardially perfused with PBS and fixed with 4% Paraformaldehyde. Brains were post-fixed for 24 hr, placed in cryoprotection (30% sucrose), and frozen at −80 °C. Frozen brains were mounted in OCT and sectioned on a Leica cryostat at 45 µm. Sections were selected at 0.85 to −0.59 mm from Bregma for S1 and IC and at −4.95 to −5.63 from Bregma for PBN based on the Mouse Brain Atlas [51]. Sections were washed in PBS, pretreated in 0.1 M Glycine (30 min at RT), washed in PBS, and incubated in SDS (10 min at RT). Sections were then washed in PBS-T, blocked in 4% NGS in PBS-T (1 hr at RT), and then incubated overnight in c-Fos antibody (Synaptic Systems, #226308; 1:2500; Goettingen, DE) in 4% NGS. Sections were then washed in PBS-T at RT, incubated with secondary antibody (2 hr at RT; Alexa Fluor 488 goat anti-guinea pig IgG; Thermo Fisher #A-11073; 1:2000), washed in PBS, and mounted on slides (VWR Superfrost Plus, 48311-703; Radnor, PA, USA) with antifade mounting medium containing DAPI (Enzo #53003; Farmingdale, NY, USA). Images were acquired at 20x magnification on an Olympus APX100 microscope system. Six images per mouse were used to quantify c-Fos positive cells within a collection of S1 and IC regions of interest and two images per mouse within the PBN regions of interest [52, 53]. The total number of positive cells per image was quantified using “GT_annotations tool” ImageJ plugin [54].

### von Frey filament test

Mice were placed on a suspended wire mesh to habituate for 1 hr the day before testing. Mice received CNO cookie dough 15 min prior to testing. The von Frey filament test consists of monofilaments of increasing diameter with forces from 0.008 to 11.0 g (North Coast Medical #NC12775-99; Morgan Hill, CA, USA). Each filament was pressed against the hind paw skin, and withdrawal/no withdrawal responses were recorded until the paw was withdrawn on 5 consecutive trials [42, 55]. The proportion of paw withdrawals during ascending trials was used to calculate the frequency of the withdrawal curve. The von Frey threshold (vF50) corresponds to the 50% frequency of withdrawal for each mouse [42].

### Home cage behavior

Mice received CNO cookie dough and were observed for 72 min with the frequency of behaviors recorded every 3 min. Recorded behaviors included eating, drinking, grooming, nest building, and the location of the behavior (in/out of nest). Home cage observations occurred within 1 hr of lights on. Chronic CNO was administered daily for 14 days with varying flavors and dosages (0.25–5 mg/kg; Table S1) [42].

### Open-field test (OFT)

Prior to testing, mice were habituated to the testing room for 30 min. The OFT consists of an open box (40 cm×40 cm x 30.5 cm), with a defined perimeter (6 inches from any wall) and a 20 ×20 cm center square. Mice were placed in the center square to start each 10 min session. Overhead digital cameras recorded center times and were calculated with EthoVision XT (Noldus, v17.5; Leesburg, VA, USA). Experiments were performed in the morning (from 0800 h to 1200 h). The OFT was cleaned with 70% EtOH between trials.

### Conditioned place preference (CPP)

The CPP test was conducted like previous DREADD-based designs [29, 30, 48]. Prior to each testing or training procedure, mice were habituated to the testing room for 30 min. On day 1, mice were allowed to explore the CPP arena (compartments: 20 cm × 20 cm x 28 cm; transition area: 19 cm × 9 cm x 28 cm with a 2.5 cm door) for 20 min. One arena contained a smooth metal floor with circular patterns on the walls and the other arena contained an angled polyurethane foam floor with stripe patterns on the walls. From days 2-6, mice were conditioned to associate a saline cookie dough treat to the preferred zone (defined during the first day) and a CNO cookie dough treat (varied flavors and doses; Table S1) to the other arena. During conditioning, mice received cookie dough ~15 min prior to conditioning in the designated compartment for 20 min. To avoid CNO remaining active between the two conditioning sessions, saline conditioning was conducted in the morning, and CNO conditioning was conducted in the afternoon. On day seven, mice were free to explore the entire arena for 20 min. The position of the animal was detected by overhead digital camera,s and arena times were calculated with EthoVision XT (Noldus, v17.5; Leesburg, VA, USA). Experiments were performed in the morning (from 0800 h to 1200 h) and afternoon (from 1300 hr to 1700 hr).

### Contextual fear conditioning and extinction

Mice were habituated to the fear conditioning chambers for 10 min (Med Associates Inc., Fairfax, VT, USA) in context A (wire grid floor cleaned with 70% EtOH), similar to previous studies [56, 57]. The next day, in context A, acquisition of the conditioned stimulus response was elicited by pairing a tone (75 dB) with a foot shock (0.4 mA) four times with a 60 sec delay between pairings. For experiment 1, extinction day 1 testing (15 tone presentations) was conducted in the same context A with CNO treats administered ~15 min prior to testing. Extinction day 2 was conducted as on day 1, but without CNO administration. For experiment 2, extinction was conducted as before, but in context B (solid metal floor cleaned with 70% EtOH plus 10% apple cider vinegar). All fear conditioning occurred in the morning (0800 h–1200 h). Freezing behavior was analyzed with ezTrack Freeze Analysis [58].

### Statistical analysis

Data are represented as mean ± SEM. All statistical analyses were performed in GraphPad Prism, (V10.4.1; Boston, MA, USA). Schematics and figures were prepared with BioRender and GraphPad Prism. Outlier analysis utilized the ROUT method (Q = 1% cutoff threshold). For all statistical tests, a value of *p* < 0.05 was considered significant. All testing was completed by experimenters blinded to treatment and genotype groups. All statistical comparisons and results are detailed in Table S2.

## Results

All statistical results are reported within the figure legends and in Table S2.

### Validation of Merkel cell-enriched keratinocyte populations

**Fig. 1:**
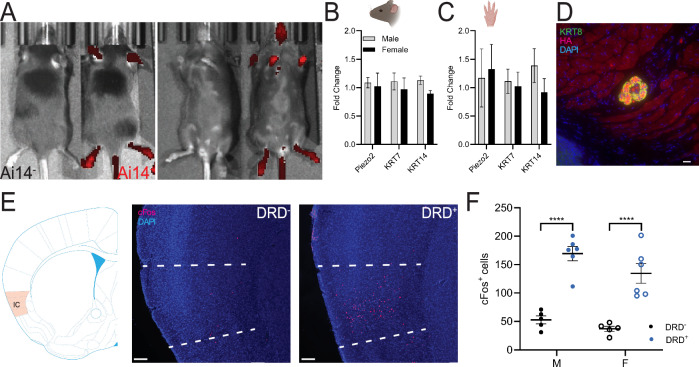
Validation of Merkel cell-enriched keratinocyte populations. **A** Dorsal (left) and ventral (right) visible skin showed robust expression of keratinocytes and Merkel cells (MC) in Ai14^+^ mice compared to Ai14^-^ littermates. **B, C** qPCR analysis confirmed expression of keratinocyte (KRT7, KRT14) and MC (KRT14, Piezo2) genes in lip and paw tissue. There is no significant sex differences for Piezo2 in lip (*n* = 4; unpaired t test: t_(6)_ = 0.3654, *p* = 0.9101) or paw (*n* = 4; unpaired t test: t_(6)_ = 0.5186, *p* = 0.7599), KRT7 in lip (*n* = 4; unpaired t test: t_(6)_ = 0.0439, *p* = 0.9102) or paw (*n* = 4; unpaired t test: t_(6)_ = 0.7002, *p* = 0.7599), or KRT14 in lip (*n* = 4; unpaired t test: t_(6)_ = 0.3654, *p* = 0.9102) or paw (*n* = 4; unpaired t test: t_(6)_ = 0.5186, *p* = 0.7599). **D** Co-labeling of KRT8 (green) and HA (red) associated with the hM3Dq transgene in DRD^+^ mice shows Gq expression in MC. Scale bar = 20 μm. **E** Representative images of c-Fos immunostaining in the Insular Cortex (IC) from DRD^-^ (left) and DRD^+^ (right) mice following stimulation of MC-enriched keratinocyte populations. Scale bar = 100μm. **F** Quantification of c-Fos positive cells in IC. DRD^+^ mice had significantly higher c-Fos positive cells compared to DRD^-^ controls with no significant difference between males and females (*n* = 5; two-way ANOVA: F_sex*genotype_(1,18) = 0.5821, *p* = 0.4554; F_sex_(1,18) = 4.046, *p* = 0.0595; F_genotype_(1,18) = 73.72, *p* < 0.0001). Ai14^+^ mice are KRT14^Cre+^ x Ai14^+^, Ai14^-^ mice are wildtype littermates. DRD^+^ mice are KRT14^Cre+^ x hM3Dq^+^, DRD^-^ mice are wildtype littermates. (****p < 0.0001).

Based on our previous findings, we aimed to validate a mouse chemogenetic model of MC-enriched keratinocyte activation in altering behaviors in adult male and female mice [14]. First, we identified KRT14^+^ cells (MC) in all visible dorsal and ventral skin using IVIS imaging of Ai14^+^ cells in KRT14^Cre^ x Ai14 mice compared to wildtype littermates (Fig. 1A). No sex differences were detected in MC-related gene expression in lips (Fig. 1B) or paws (Fig. 1C) of wildtype mice [26, 59]. Individual channel images of DAPI (Fig. S1A), α-KRT8 (Fig. S1B), and HA (Fig. S1C) reveal the robust expression of hM3dq DREADD and the α-KRT8 MC marker. Co-labeling of α-KRT8 (MCs) and HA (DREADD hM3Dq) in DRD^+^ mice shows DREADD Gq expression in KRT8 + MC (Fig. 1D and Fig. S1D).

We next identified cortical activation of neurons in KRT14^Cre+^ x hM3Dq^+^ (DRD^+^) mice compared to wildtype littermates (DRD^-^). Following CNO administration, the number of c-Fos positive cells was significantly higher in the insular cortex (IC) of male and female DRD^+^ mice (Fig. 1E, F). Additionally, c-Fos positive cell counts were increased in the somatosensory cortex (S1) and parabrachial nucleus (PBN) of male and female DRD^+^ mice (Fig. S1E, F). We did not detect sex differences in c-Fos positive cell counts in the IC or S1 following CNO administration. DRD^+^ male mice had more c-Fos positive cells in the PBN compared to DRD^+^ females (Fig. S1G, H).

### Merkel cell stimulation does not alter tactile sensitivity, but does increase grooming behavior and codes for negative valence

**Fig. 2:**
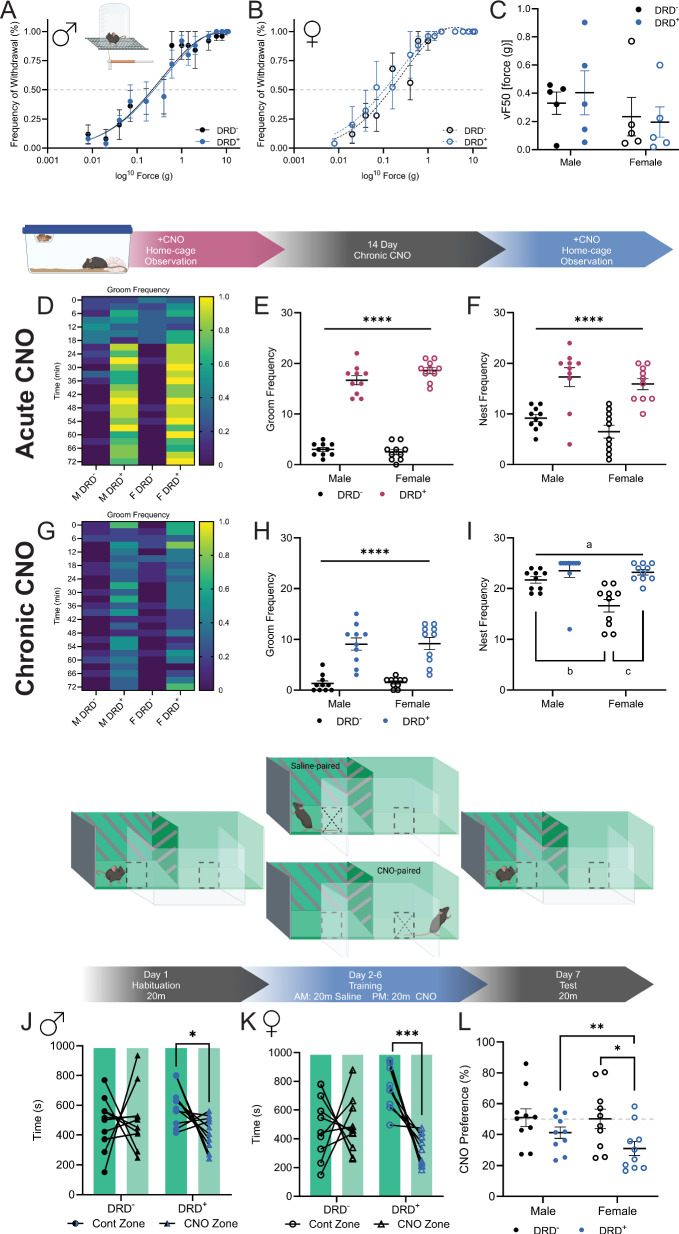
Tactile sensitivity, home-cage behavior, and valence following MC stimulation. The von Frey filament test was used to measure tactile sensitivity. The paw withdrawal curve was not different in male (**A**; Nonlinear fit: *n* = 5; F_(1,72)_ = 0.08362, *p* = 0.9689) or female (**B**; Nonlinear fit: *n* = 5; F_(1,72)_ = 0.4887, *p* = 0.6907) DRD^+^ mice, compared to DRD^-^ littermates following MC stimulation. **C** There was also no difference in vF50 for tactile sensitivity following MC stimulation (*n* = 5; two-way ANOVA: F_sex*genotype_(1,16) = 0.0578, *p* = 0.8131; F_sex_(1,16) = 0.1361, *p* = 0.7171; F_genotype_(1,16) = 2.215, *p* = 0.1561). Home-cage observations were conducted following the first dose of CNO. **D, E** Grooming frequency was increased in DRD^+^ mice compared to DRD^-^ littermates following the initial MC stimulation independent of sex (*n* = 10; two-way ANOVA: F_sex*genotype_(1,36) = 3.527, *p* = 0.0685; F_sex_(1,36) = 1.2, *p* = 0.2806; F_genotype_(1,36) = 543.7, *p* < 0.0001). **F** The total nest frequency was also increased in DRD^+^ mice compared to DRD^-^ littermates after MC stimulation (*n* = 10; two-way ANOVA: F_sex*genotype_(1,36) = 0.2489, *p* = 0.6209; F_sex_(1,36) = 2.476, *p* = 0.1244; F_genotype_(1,36) = 45.1, *p* < 0.0001). **G**, **H** Following 14 d chronic CNO, grooming frequency was still increased in DRD^+^ mice compared to DRD^-^ littermates (*n* = 10; two-way ANOVA: F_sex*genotype_(1,36) = 0.003, *p* = 0.9566; F_sex_(1,36) = 0.0271, *p* = 0.8702; F_genotype_(1,36) = 72.29, *p* < 0.0001) following MC stimulation. **I** Total nest frequency was also increased in DRD^+^ mice following 14 d chronic CNO, compared to DRD^-^ littermates (*n* = 10; two-way ANOVA^:^ F_sex*genotype_(1,36) = 5.881, *p* = 0.0205; F_sex_(1,36) = 7.443, *p* = 0.0098; a=F_genotype_(1,36) = 18.01, *p* = 0.0001). Female DRD^-^ mice spent less time in the nest than male DRD^-^ mice (b = Tukey post-hoc *p* = 0.0045^)^ and female DRD^+^ mice (c = Tukey post-hoc *p* = 0.0002^)^. **J** Conditioned place preference revealed a negative valence of MC stimulation. Male DRD^-^ mice did not show a preference for either control or CNO-paired arenas (*n* = 10; paired t test: t_(9)_ = 0.2522, *p* = 0.8065). Male DRD^+^ mice preferred the control-paired arena compared to the CNO-paired arena (*n* = 10; paired t test: t_(9)_ = 2.410, *p* = 0.0393). **K** Female DRD^-^ mice did not show a preference for either control or CNO-paired arenas (*n* = 10; paired t test: t_(9)_ = 0.0968, *p* = 0.9520). Female DRD^+^ mice preferred the control-paired arena compared to the CNO-paired arena (*n* = 10; paired t test: t_(9)_ = 5.632, *p* = 0.0003). **L** Overall, the preference for the CNO-paired arena (%) was decreased in male and female DRD^+^ mice (*n* = 10; two-way ANOVA: F_sex_*_genotype_(1,36) = 0.8244, *p* = 0.3699; F_sex_(1,36) = 1.138, *p* = 0.2932; a=F_genotype_(1,36) = 7.797, *p* = 0.0083) compared to DRD^-^ mice; especially in female DRD^+^ compared to female DRD^-^ mice (b=Tukey post-hoc *p* = 0.0129). DRD^+^ mice are KRT14^Cre+^ x hM3Dq^+^, DRD^-^ mice are wildtype littermates. (*p < 0.05, ****p < 0.0001).

To identify the influence of MC stimulation on tactile sensitivity, we utilized the von Frey filament test. There was no effect of genotype on paw withdrawal curves following CNO treatment for male (Fig. 2A) or female (Fig. 2B) mice. Similarly, von Frey thresholds (vF50) were not influenced by MC stimulation in either male or female mice of either genotype (Fig. 2C). No sex differences were detected in paw withdrawal curves or vF50.

Home cage observations were conducted following the first CNO dose and after two weeks of daily CNO dosing to examine the effect of MC stimulation on naturalistic behaviors. After the first CNO dose, male and female DRD^+^ mice spent significantly more time grooming (Fig. 2D, E and supplemental video 1) and more time in their nest (Fig. 2F) than DRD^-^ littermates (supplemental video 2). DRD^+^ mice also spent more time grooming in their nest (Fig. S2A), had a higher ratio of in nest grooming: in nest time (Fig. S2B), and lower frequency of all other observed behaviors (Fig. S2C). To examine if these effects were due to novelty of MC stimulation, we examined these behaviors following two weeks of daily CNO. DRD^+^ mice maintained a similar response to MC stimulation, with both males and females spending more time grooming than DRD^-^ littermates (Fig. 2G, H). Total nest frequency revealed an interaction, driven by decreased nest time in DRD^-^ female mice compared to male DRD^-^ mice and female DRD^+^ mice (Fig. 2I). Compared to DRD^-^ littermates, DRD^+^ male and female mice also spent significantly more time grooming in their nest (Fig. S2D), had an increased ratio of in nest grooming: in nest time (Fig. S2E), and had a lower frequency of all other observed behaviors, including feeding, drinking, and nest building, (Fig. S2F). These responses do not appear to be driven exclusively by an anxiety-like response, as there was no difference in OFT center time following acute (~10 min) CNO stimulation of MC (Fig. S2G).

To determine the valence of MC stimulation, we conducted conditioned place preference (CPP). Following training, both male (Fig. 2J) and female (Fig. 2K) DRD^+^ mice spent significantly more time in the saline-paired arena compared to the CNO-paired arena. DRD^-^ mice did not display an arena preference following training. The percentage of time and difference in time spent in the CNO-paired arena was decreased in DRD^+^ male and female mice (Fig. 2L, S2H). While patterns in the data suggest potential for sex differences in magnitude of effects, no interaction between sexes was identified. Future studies should dissect unique aspects of sex specific central integration of this sensory stimulation.

### Merkel cell stimulation attenuates fear extinction

**Fig. 3:**
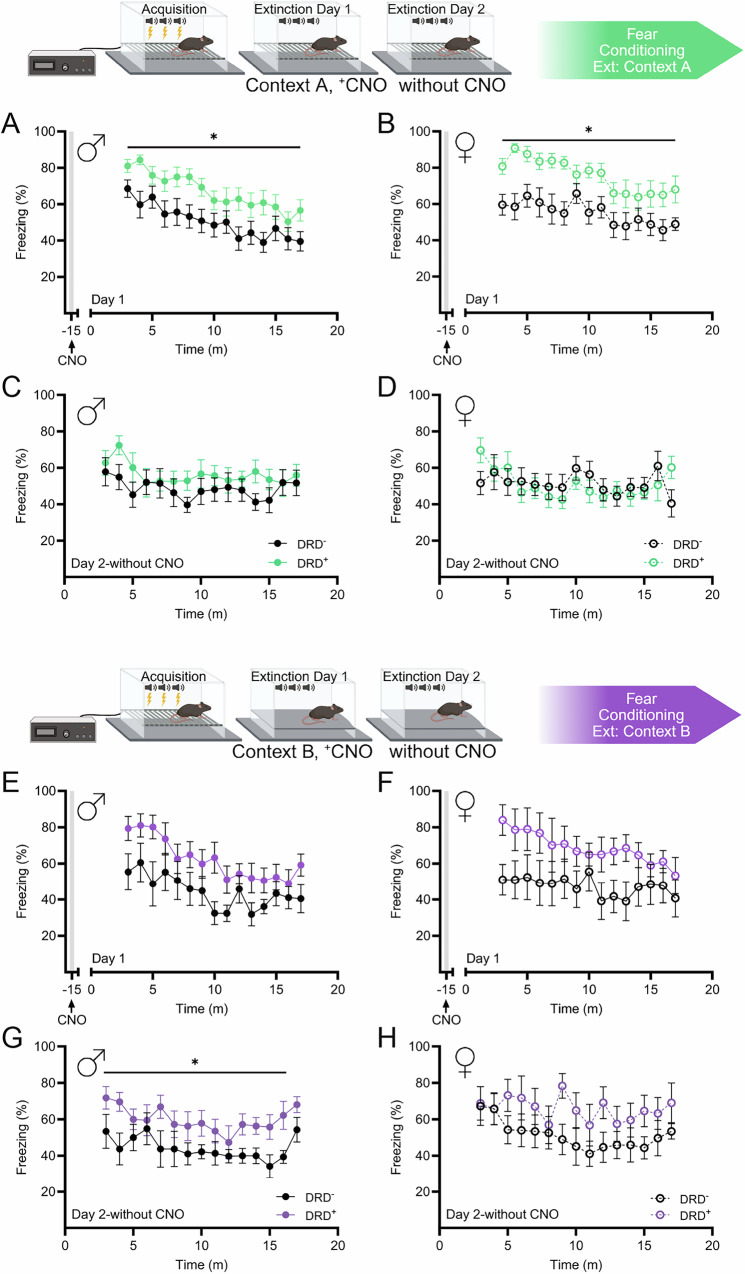
Fear extinction is dependent on MC stimulation. **A** Male DRD^+^ mice spent more time freezing following CNO, on extinction day 1 (*n* = 15; two-way RM ANOVA: F_time*genotype_(14, 392) = 0.6724, *p* = 0.8016; F_time_(5.856, 164) = 10.03, *p* < 0.0001; F_genotype_(1, 28) = 6.677, *p* = 0.0153). **B** Female DRD^+^ mice also spent more time freezing with CNO, on extinction day 1 (*n* = 14; two-way RM ANOVA: F_time*genotype_(14, 364) = 0.9085, *p* = 0.5498; F_time_(4.86, 126.4) = 6.12, *p* < 0.0001; F_genotype_(1, 26) = 10.81, *p* = 0.0029). **C** On extinction day 2, without CNO, there was no difference in freezing behavior in male mice (*n* = 15; two-way RM ANOVA: F_time*genotype_(14, 252) = 0.9439, *p* = 0.5122; F_time_(7.535, 135.6) = 2.077, *p* = 0.0456; F_genotype_(1, 18) = 1.142, *p* = 0.2994). **D** On extinction day 2, without CNO, DRD^+^ female mice had different freezing patterns than DRD^-^ mice (n = 14; two-way RM ANOVA: F_time*genotype_(14, 196) = 1.911, *p* = 0.0272; F_time_(14, 196) = 2.258, *p* = 0.0072; F_genotype_(1, 14) = 0.0066, *p* = 0.9664). **E** In context B, on day 1 with CNO, male DRD^+^ and WT spent similar amounts of time freezing (two-way RM ANOVA: *n* = 8; F_time*genotype_(14, 210) = 1.091, *p* = 0.367; F_time_(4.313, 64.69) = 7.786, *p* < 0.0001; F_genotype_(1, 15) = 4.012, *p* = 0.0636). **F** Female mice did not show significantly different freezing behavior in context B on day 1, with CNO (two-way RM ANOVA: *n* = 7; F_time*genotype_(14, 154) = 0.8588, *p* = 0.6046; F_time_(4.131, 45.44) = 2.106, *p* = 0.0935; F_genotype_(1, 11) = 2.999, *p* = 0.1112). **G** On extinction day 2, without CNO, male DRD^+^ mice spent more time freezing compared to WT littermates (two-way RM ANOVA: *n* = 8; F_time*genotype_(14, 210) = 0.6007, *p* = 0.8629; F_time_(6.537, 98.05) = 2.189, *p* = 0.0454; F_genotype_(1, 15) = 6.413, *p* = 0.023). **H** Female mice did not spend more time freezing on extinction day 2, without CNO (two-way RM ANOVA: *n* = 7; F_time*genotype_(14, 168) = 1.108, *p* = 0.3538; F_time_(14, 168) = 2.177, *p* = 0.0105; F_genotype_(1, 12) = 1.965, *p* = 0.1863). DRD^+^ mice are KRT14^Cre+^ x hM3Dq^+^, DRD^-^ mice are wildtype littermates. (*p < 0.05).

Given the potential for MC stimulation to alter tactile perception, we conducted fear conditioning with extinction paradigms occurring in different tactile and odor settings. On extinction day 1, with CNO in context A, both male (Fig. 3A) and female (Fig. 3B) DRD^+^ mice displayed increased freezing compared to DRD^-^ littermates. However, on extinction day 2, without CNO, freezing time was not significantly different between DRD^+^ male or female mice and their DRD^-^ littermates (Fig. 3C, D). To identify the impact of context and tactile perception on fear extinction, we also conducted extinction trials in context B. On extinction day 1 with CNO, while a similar pattern of increased freezing appears for DRD^+^ male (Fig. 3E) and female (Fig. 3F) mice, neither group reached statistical significance compared to DRD^-^ littermates. On extinction day 2 without CNO, the increased freezing in male DRD^+^ mice compared to DRD^-^ littermates was statistically significant (Fig. 3G), but a similar pattern of differences did not reach statistical significance in female mice (Fig. 3H). AUC analyses for extinction curves revealed similar patterns; increased freezing in both male and female DRD^+^ mice on extinction day 1, with CNO in context A and increased freezing only in male DRD^+^ mice on extinction day 2, without CNO in context B (Fig. S2I, J).

## Discussion

Recent studies have identified a significant impact of stress and trauma on sensory pathways [4, 5, 60–63]. We previously identified keratinocyte and MC-related protein expression in an unbiased proteomics screen from circulating extracellular vesicles in adult women who had experienced sexual trauma [14]. Sexual trauma alters insular and somatosensory cortices, as well as heightened fear conditioning, however, a preclinical model to assess the underlying mechanisms of sensory involvement in fear memory processing has not been developed [5, 17, 18, 64]. Therefore, we utilized chemogenetic activation of MC in mice as a model to assess potential MC involvement in specific fear and stress behavioral assays.

First, to validate this model, we examined expression patterns of the KRT14^cre^ utilizing in vivo imaging system (IVIS) and an Ai14 fluorescent reporter to visualize MC-enriched populations of keratinocytes [24, 43, 45, 46, 65, 66]. IVIS imaging revealed fluorescence in KRT14^+^ cells, including lips, ears, paws, genitals, and tail, consistent with expected MC expression [20, 21, 24, 37, 39, 43, 45, 46, 67]. Additionally, we compared expression levels of known genes important to MC differentiation and function in lips and paws from male and female mice using real-time PCR. We did not detect sex differences in the levels of pre- and post-differentiation keratinocyte (KRT7 and KRT14) and MC (KRT14 and Piezo2) related genes [26, 59]. Additionally, co-labeling of the hM3Dq DREADD receptor HA with the TROMA-I α-KRT8 confirmed the presence of hM3Dq DREADD expression in MC. Combined, these results validate that our KRT14^Cre^ mouse line expresses in specific MC skin areas and that these tissues express relevant MC marker genes at similar levels between male and female mice.

Next, we focused on the CNS to identify the impact of MC activation on neural activity in DREADD positive (DRD^+^) mice as evidence that CNO treatment could produce neuronal activation within the insular cortex (IC) based on its role in somatosensory and valence perception [30, 40, 41, 68, 69]. As expected, male and female DRD^+^ mice showed significantly increased c-Fos positive cells in IC compared to DRD^-^ controls following CNO treatment. Additionally, MC stimulation increased c-Fos-positive cells in the S1 and PBN [53, 68, 70–72]. MCs form a synapse at the Merkel disc in touch domes projecting to the dorsal root ganglion via Aβ-afferents [22, 26, 73–75]. C-LTMR (C-fiber low threshold mechanoreceptor) afferents are largely responsible for ‘emotional touch’, however, significant overlap exists in the distribution of Aβ- and C-LTMRs in hairy skin, their innervation of hair follicles (C-LTMRs are not present in glabrous skin), and the brain regions that they activate [30, 40, 41, 53, 76–82]. While activation of Aβ-afferents is well defined, our study demonstrates the novel identification of IC neuronal activation following MC stimulation [22–26, 43–46]. Interestingly, beyond its role in affiliative touch processing, the IC is highly involved in valence detection and the sensory integration of fear signals [52, 69, 72, 83–88].

Based on the role of MC in the detection of light touch, we next validated the role of acute MC stimulation on tactile sensitivity [19–21]. To identify the influence of MC activation on local tactile sensitivity, we tested mice in the von Frey monofilament test and found no genotypic differences in paw withdrawal following CNO treatment. Previous studies did not identify tactile differences with chemogenetic or optogenetic stimulation of MC-enriched populations of keratinocytes, but describe increased frequencies of grooming or itching [43–46, 65, 66]. Interestingly, decreased tactile sensitivity and reduced Aβ-afferent responses in mice with MC-specific Piezo2 knockout or optogenetic inhibition have been reported [23–25, 89].

As acute CNO stimulation did not alter tactile sensitivity, we examined mice for a behavioral confirmation that they were detecting CNO stimulation of MC. In a home cage behavioral assessment following acute CNO administration, male and female DRD^+^ mice performed significantly more grooming behavior in their nest compared to DRD^-^ littermates. Based on the significant increase in time spent grooming, not surprisingly DRD^+^ mice spent less time engaging in other naturalistic behaviors. The grooming phenotype aligns with previous reports of chemogenetic and optogenetic stimulation of MC and a potential role of MC involvement in converting the perception of touch to itch [43–46, 65].

To determine if these effects of CNO on grooming were due to the novelty of acute MC stimulation, we also examined mice following daily CNO treatment. Following 14 days of CNO administration, male and female DRD^+^ mice still performed grooming behavior at a significantly higher rate than DRD^-^ mice, while also performing fewer other naturalistic behaviors, including feeding, drinking, and nest building. However, the effect was less robust following the chronic CNO administration and suggests that mice do partially habituate to MC stimulation. The persistence of the grooming phenotype (~60 min) in DRD^+^ mice indicates a potentially aversive or stressful perception of MC stimulation associated with IC activation, despite the lack of an effect of MC stimulation in the OFT [90–93].

We next wanted to identify the valence of MC stimulation. To quantify positive or negative valence, we conducted a conditioned place preference test. DRD^+^ mice displayed a significant avoidance response to the CNO-paired arena compared to the saline-paired arena; an effect not found in the DRD^-^ mice. Additionally, this effect was greater in DRD^+^ female mice compared to males, suggesting potential sex differences in the intensity of CNO stimulation of MC or central integration of this sensory perception. Previous studies have identified sex differences in preference or valence responses to emotional stimuli that are associated with IC activity [85, 86, 94–96]. Interestingly, while we found that MC stimulation produced a CNO-paired aversion, previous studies found that C-LTMR stimulation was associated with CNO-paired preference [29, 30, 48]. These opposing behavioral outcomes may reflect the heterogeneity of cell types and subregions within the IC that are activated by ascending Aβ and C-LTMR pathways [30, 40, 41, 52, 68, 77–82, 85, 86]. Overall, this data aligns with previous reports of changes in IC activity during conditioned aversion, with negative valence, and following stress or trauma [97–102].

Changes in sensory systems have been described in PTSD, and survivors of sexual trauma display decreased activation of the somatosensory and insular cortices [2–10, 17, 18]. Additionally, violent or sexual trauma is associated with heightened fear conditioning and deficits in fear extinction [11–16, 64]. These effects are often stronger in women [13, 14, 16, 103]. While sex differences have not been directly examined in mechanosensory systems, RNA sequencing of Aβ-afferent containing dorsal root ganglia neurons has identified sex-specific gene expression differences [104–106]. To this end, we next validated our MC stimulation model in a PTSD-relevant fear conditioning task. We found that CNO administration prior to extinction day 1 produced increased freezing behavior, indicating an augmented fear response in both male and female DRD^+^ mice. However, in the absence of further CNO treatment on day 2, extinction in both male and female mice was indistinguishable from DRD^-^ littermates, suggesting that this effect might be reflective of a heightened sensory sensitivity following acute MC activation that impacts extinction in this same context.

Next, to examine the potential role of context in extinction learning following MC activation, we examined mice following acute CNO treatment. Again, similar to context A, when CNO was administered prior to fear extinction day 1 but in a novel context (context B), we saw a similar pattern as previously with increased freezing responses in DRD^+^ male and female mice. However, with the increased variance in both genotypes in this novel context, these differences did not reach statistical significance, likely due to the stress of novelty for all mice or a potential floor effect of freezing in mice [107]. Interestingly, on the second extinction day, in context B, male DRD^+^ mice reached significance for increased freezing, potentially due to pairing context B with CNO administration on extinction day one, leading to reduced variance on the second extinction day. Female mice showed similar increased freezing on day 2, but again, this did not reach significance. We note that the lack of sex differences in fear extinction is surprising, despite females showing a larger negative valence effect in the conditioned place preference. This could be explained by an increased stress sensitivity to the novel context for both DRD^+^ and DRD^-^ female mice [108–113]. Combined, these findings validate a role for MC sensory activation in the expression of fear memory and identify exciting targets of future studies, especially known circuits between the somatosensory cortex, insular cortex, prefrontal cortex, and amygdala [114–121].

Increased fear-related responses in female rodents and risk for developing PTSD in women have been previously reported [94–96, 110, 113, 122–126]. Meta-analyses report significant sex differences not only in PTSD vulnerability, but also for differences in the experience of the traumatizing event and the underlying neural activation in response to stimuli with positive and negative emotional valence [94–96, 117, 122, 124]. Further, biological differences in fear responding may be influenced by sex hormones and an evolutionary ability to differentially adapt to stress or trauma [12, 96, 124, 127, 128]. Ongoing studies should focus on mechanistic questions to resolve the neural and biological underpinnings of MC function as they pertain to sex differences in valence perception and conditioned fear. Finally, as MCs reach full developmental differentiation during critical pubertal windows, a period that also represents increased vulnerability to trauma, future studies can utilize preclinical models to focus on the impact of stress and MC activation during these developmental periods [14, 78, 103, 129–134]. These studies support an opportunity to utilize a preclinical model to explore potential sensory-based therapeutic options for anxiety and PTSD-related symptoms [135–143].

## Supplementary information

Supplemental Video 1

Supplemental Video 2

Supplemental Table 1

Supplemental Table 2

Supplemental Figure 1

Supplemental Figure 2

Supplemental Figure Legends

## Data Availability

All relevant data are available upon response request to the authors.
