# Supplementary material for: Merkel cell stimulation in fear and sensory signaling
Source: Neuropsychopharmacology. 2025 Jun 7;50(9):1395–405. doi: 10.1038/s41386-025-02144-w (PMC12260070; doi:10.1038/s41386-025-02144-w)
Supplement: Supplementary file 3 — Supplemental Table 1 [file 41386_2025_2144_MOESM3_ESM.docx]

| Day | Dose (mg/kg) | Flavor | Test Details |
| --- | --- | --- | --- |
| 1 | 5 | Bacon | Home Cage Observation |
| 2 | .25 | Sugar | CPP Habituation (Dough after Test) |
| 3 | .5 | Peanut Butter | CPP training |
| 4 | 1 | Nutella | CPP training |
| 5 | .5 | Bacon | CPP training |
| 6 | 1 | Peanut Butter | CPP training |
| 7 | .25 | Bacon | CPP Test (Dough after Test |
| 8 | .5 | Nutella | N/A |
| 9 | 1 | Bacon | N/A |
| 10 | .5 | Peanut Butter | N/A |
| 11 | .25 | Nutella | N/A |
| 12 | .5 | Sugar | N/A |
| 13 | 1 | Nutella | N/A |
| 14 | 5 | Bacon | Home Cage Observation |
