# Supplementary material for: Merkel cell stimulation in fear and sensory signaling
Source: Neuropsychopharmacology. 2025 Jun 7;50(9):1395–405. doi: 10.1038/s41386-025-02144-w (PMC12260070; doi:10.1038/s41386-025-02144-w)
Supplement: Supplementary file 4 — Supplemental Table 2 [file 41386_2025_2144_MOESM4_ESM.docx]

| Figure | n | Test | Factors | Post-hoc Testing (when appropriate) | F_(DFn, DFd)_ or t_(df)_ | P value |
| --- | --- | --- | --- | --- | --- | --- |
| 1B_Piezo2_ | 4 | t test | sex | Holm-Šídák method | t=0.3654_(6)_ | 0.9101 |
| 1B_KRT7_ | 4 | t test | sex | Holm-Šídák method | t=0.0439_(6)_ | 0.9102 |
| 1B_KRT14_ | 4 | t test | sex | Holm-Šídák method | t=0.3654_(6)_ | 0.9102 |
| 1C_Piezo2_ | 4 | t test | sex | Holm-Šídák method | t=0.5186_(6)_ | 0.7599 |
| 1C_KRT7_ | 4 | t test | sex | Holm-Šídák method | t=0.7002_(6)_ | 0.7599 |
| 1C_KRT14_ | 4 | t test | sex | Holm-Šídák method | t=0.5186_(6)_ | 0.7599 |
| 1E | 5 | 2-way ANOVA | sex x genotype | Tukey’s multiple comparisons | Interaction: F=0.5821_(1,18)_  Sex: F=4.0460_(1,18)_  Genotype: F=73.72_(1,18)_ | 0.4554  0.0595  <0.0001 |
| 2A | 5 | nonlinear fit | genotype | none | F=0.8362_(1,72)_ | 0.9689 |
| 2B | 5 | nonlinear fit | genotype | none | F=0.4887_(1,72)_ | 0.6907 |
| 2C | 5 | 2-way ANOVA | sex x genotype | Tukey’s multiple comparisons | Interaction F=0.0578_(1,36)_  Sex F=0.1361_(1,36)_  Genotype F=2.215_(1,36)_ | 0.8131  0.7171  0.1561 |
| 2E | 10 | 2-way ANOVA | sex x genotype | Tukey’s multiple comparisons | Interaction F=3.527_(1,36)_  Sex F=1.2_(1,36)_  Genotype F=543.7_(1,36)_ | 0.0685  0.2806  <0.0001 |
| 2F | 10 | 2-way ANOVA | sex x genotype | Tukey’s multiple comparisons | Interaction F=0.2489_(1,36)_  Sex F=2.476_(1,36)_  Genotype F=45.1_(1,36)_ | 0.6209  0.1244  <0.0001 |
| 2H | 10 | 2-way ANOVA | sex x genotype | Tukey’s multiple comparisons | Interaction F=0.003_(1,36)_  Sex F=0.0271_(1,36)_  Genotype F=72.29 _(1,36)_ | 0.9566  0.8702  <0.0001 |
| 2I | 10 | 2-way ANOVA | sex x genotype | Tukey’s multiple comparisons | Interaction F=5.881_(1,36)_  Sex F=7.443_(1,36)_  Genotype F=18.01_(1,36)_ | 0.0205  0.0098  0.0001 |
| 2J | 10 | t test | arena | none | DRD^-^ t=0.2522_(9)_  DRD^+^ t=2.410_(9)_ | 0.8065  0.0393 |
| 2K | 10 | t test | arena | none | DRD^-^ t=0.0968_(9)_  DRD^+^ t=5.632_(9)_ | 0.9520  0.0003 |
| 2L | 10 | 2-way ANOVA | sex x genotype | Tukey’s multiple comparisons | Interaction F=0.8244_(1,36)_  Sex F=1.138_(1,36)_  Genotype F=7.797_(1,36)_ | 0.3699  0.2932  0.0083 |
| 3A | 15 | 2-way RM ANOVA | time x genotype | none | Interaction F=0.8244_(14,392)_  Time F=10.03_(5.856, 164)_  Genotype F=7.797_(1, 28)_ | 0.8016  <0.0001  0.0153 |
| 3B | 14 | 2-way RM ANOVA | time x genotype | none | Interaction F=0.9085_(14,364)_  Time F=6.12_(4.86, 126.4)_  Genotype F=10.81 _(1, 26)_ | 0.5498  <0.0001  0.0029 |
| 3C | 15 | 2-way RM ANOVA | time x genotype | none | Interaction F=0.9439_(14, 252)_  Time F=2.077_(7.535, 135.6)_  Genotype F=1.142_(1, 18)_ | 0.5122  0.0456  0.2994 |
| 3D | 14 | 2-way RM ANOVA | time x genotype | none | Interaction F=1.911_(14,196)_  Time F=2.258_(14, 196)_  Genotype F=0.0066_(1, 14)_ | 0.0272  0.0072  0.9664 |
| 3E | 8 | 2-way RM ANOVA | time x genotype | none | Interaction F=1.091_(14,210)_  Time F=7.786_(4.313, 64.69)_  Genotype F=4.012_(1, 15)_ | 0.367  <0.0001  0.0636 |
| 3F | 7 | 2-way RM ANOVA | time x genotype | none | Interaction F=0.8588_(14, 154)_  Time F=2.106_(4.131, 45.44)_  Genotype F=2.99_(1, 11)_ | 0.6046  0.0935  0.1112 |
| 3G | 8 | 2-way RM ANOVA | time x genotype | none | Interaction F=0.6007_(14,210)_  Time F=2.189_(6.537, 98.05)_  Genotype F=6.413_(1, 15)_ | 0.8629  0.0454  0.023 |
| 3H | 7 | 2-way RM ANOVA | time x genotype | none | Interaction F=1.108_(14, 168)_  Time F=2.177_(14, 168)_  Genotype F=1.965_(1, 12)_ | 0.3538  0.0105  0.1863 |
| S1A | 10 | 2-way ANOVA | sex x genotype | Tukey’s multiple comparisons | Interaction F=1.598_(1,36)_  Sex F=0.000_(1,36)_  Genotype F=154 _(1,36)_ | 1.598  >0.999  <0.0001 |
| S1B | 10 | 2-way ANOVA | sex x genotype | Tukey’s multiple comparisons | Interaction F=1.353_(1,36)_  Sex F=1.693_(1,36)_  Genotype F=59.72_(1,36)_ | 0.2524  0.2014  <0.0001 |
| S1C | 10 | 2-way ANOVA | sex x genotype | Tukey’s multiple comparisons | Interaction F=1.497_(1,36)_  Sex F=1.124_(1,36)_  Genotype F=8.149_(1,36)_ | 0.2291  0.2961  0.0071 |
| S1D | 10 | 2-way ANOVA | sex x genotype | Tukey’s multiple comparisons | Interaction F=0.0793_(1,36)_  Sex F=0.0793_(1,36)_  Genotype F=74.21_(1,36)_ | 0.7799  0.7799  <0.0001 |
| S1E | 10 | 2-way ANOVA | sex x genotype | Tukey’s multiple comparisons | Interaction F=0.0436_(1,36)_  Sex F=2.0032_(1,36)_  Genotype F=70.71_(1,36)_ | 0.8357  0.9551  <0.0001 |
| S1F | 10 | 2-way ANOVA | sex x genotype | Tukey’s multiple comparisons | Interaction F=0.0063_(1,36)_  Sex F=0.3095_(1,36)_  Genotype F=6.069 _(1,36)_ | 0.9371  0.5814  0.0187 |
| S1G | 10 | 2-way ANOVA | sex x genotype | Tukey’s multiple comparisons | Interaction F=1.305_(1,36)_  Sex F=2.411_(1,36)_  Genotype F=10.31_(1,36)_ | 0.2609  0.1292  0.0028 |
