# Supplementary figures and images for: Merkel cell stimulation in fear and sensory signaling
Source: Neuropsychopharmacology. 2025 Jun 7;50(9):1395–405. doi: 10.1038/s41386-025-02144-w (PMC12260070; doi:10.1038/s41386-025-02144-w)

# Supplemental Figure 1

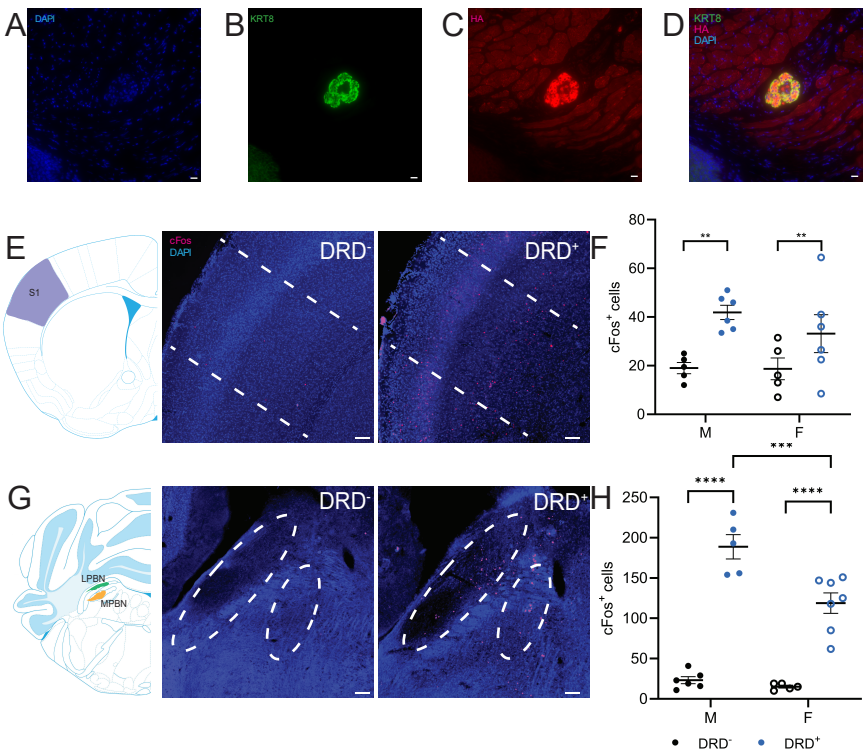

Supplement: Supplementary file 5 — Supplemental Figure 1 [file 41386_2025_2144_MOESM5_ESM.pdf]

# Supplemental Figure 2

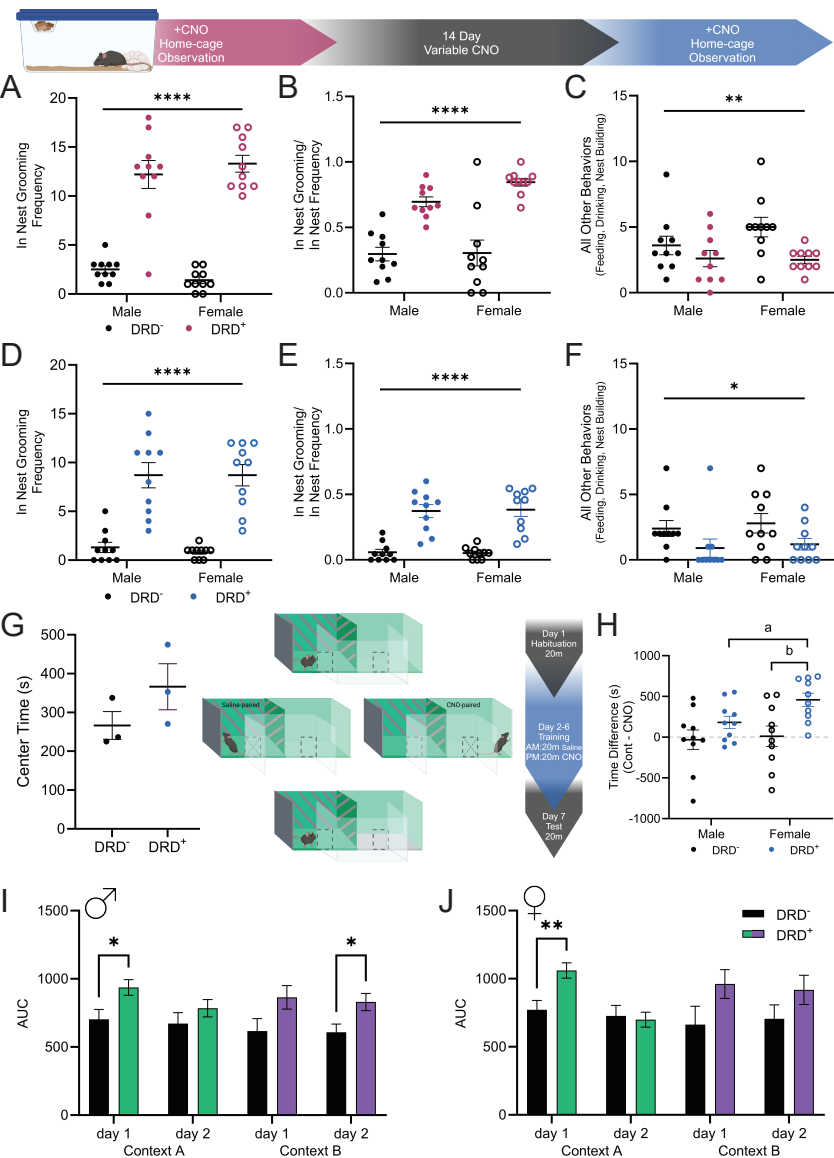

Supplement: Supplementary file 6 — Supplemental Figure 2 [file 41386_2025_2144_MOESM6_ESM.pdf]
