# Supplementary material for: Merkel cell stimulation in fear and sensory signaling
Source: Neuropsychopharmacology. 2025 Jun 7;50(9):1395–405. doi: 10.1038/s41386-025-02144-w (PMC12260070; doi:10.1038/s41386-025-02144-w)
Supplement: Supplementary file 7 — Supplemental Figure Legends [file 41386_2025_2144_MOESM7_ESM.docx]

**Figure S1. Merkel cell, somatosensory cortex, and parabrachial nucleus immunofluorescence A** DAPI staining of fixed paw tissue from DRD^+^ mouse. **B** TROMA-I α-Krt8 labeling of fixed paw tissue from DRD^+^ mouse. **C** HM3DQ DREADD-associated HA in fixed paw tissue from DRD^+^ mouse. **D** Co-labelling of KRT8 (green) and HA (red) associated with the hM3Dq transgene in DRD^+^ mice shows Gq expression in MC. Scale bar = 20 μm. **E** Representative images of c-Fos immunostaining in the S1 Cortex from DRD^-^ (left) and DRD^+^ (right) mice. Scale bar = 100 μm. **F** Quantification of c-Fos positive cells in S1 cortex. DRD^+^ mice had significantly higher c-Fos positive cells compared to DRD^-^ controls with no significant difference between males and females (n=5; two-way ANOVA: F_sex*genotype_(1,18)=0.6796, *p*=0.4205; F_sex_(1,18)=0.7795, *p*=0.3889; F_genotype_(1,18)=13.30, *p*=0.0018). **G** Representative images of c-Fos immunostaining in the lateral and medial parabrachial nucleus (LPBN and MPBN) from DRD^-^ (left) and DRD^+^ (right) mice. Scale bar = 100μm. **H** Quantification of c-Fos positive cells in the PBN. DRD^+^ mice had significantly higher c-Fos positive cells compared to DRD^-^ controls with no significant difference between males and females (n=5-7; two-way ANOVA: F_sex*genotype_(1,19)=8.543, *p*=0.0087; F_sex_(1,19)=13.5, *p*=0.3889; F_genotype_(1,19)=161.3, *p*<0.0001).

**Figure S2. Home-cage behavior and valence following MC stimulation. A** The occurrence of grooming in the nest was increased in DRD^+^ mice compared to DRD^-^ littermates following the initial MC stimulation (n=10; F_sex*genotype_(1,36)=1.598, *p=*0.2143; F_sex_(1,36)=0.000, p>0.999; F_genotype_(1,36)=154.0, *p<*0.0001). **B** The ratio of in nest grooming to overall time in nest was also increased in DRD^+^ mice compared to DRD^-^ prior to chronic CNO (n=10; F_sex*genotype_(1,36)=1.353, *p=*.2524; F_sex_(1,36)=1.693, *p=*0.2014; F_genotype_(1,36)=59.72, *p<*0.0001). **C** The occurrence of all other behaviors was decreased in DRD^+^ mice compared to DRD^-^ prior to (n=10; F_sex*genotype_(1,36)=1.497, *p=*0.2291; F_sex_(1,36)=1.124, *p=*0.2961; F_genotype_(1,36)=8.149, *p=*0.0071). **D** The occurrence of grooming in the nest was also increased in DRD+ mice following 14d CNO compared to DRD- mice (n=10; F_sex*genotype_(1,36)=0.0793, *p=*0.7799; F_sex_(1,36)=0.0793, *p=*0.7799; F_genotype_(1,36)=74.21, *p<*0.0001). **E** The ratio of in nest grooming to overall time in nest was also increased in DRD^+^ mice compared to DRD^-^ prior to chronic CNO following 14 d CNO (n=10; F_sex*genotype_(1,36)=0.0436, *p=*0.8357; F_sex_(1,36)=2.0032, *p=*0.9551; F_genotype_(1,36)=70.71, *p<*0.0001). **F** The occurrence of all other behaviors was decreased in DRD^+^ mice compared to DRD^-^ following 14 d CNO (n=10; F_sex*genotype_(1,36)=0.0063, *p=*0.9371; F_sex_(1,36)=0.3095, *p=*0.5814; F_genotype_(1,36)=6.069, *p=*0.0187). **G** Center time in the open-field test was not impacted by CNO in DRD+ mice, compared to DRD- littermates (n=3; unpaired t test: t(4)=1.444, p=0.2224). **H** The total difference in time spent in each arena (Cont-CNO) was greater in DRD^+^ mice compared to DRD^-^ littermates following MC stimulation (n=10; F_sex*genotype_(1,36)=1.305, *p=*0.2609; F_sex_(1,36)=2.411, *p=*0.1292; F_genotype_(1,36)=10.31, *p=*0.0028). **I** AUC analysis revealed increased freezing in DRD^+^ male mice on extinction day 1 with CNO in context A and on day 2 without CNO in context B (n=9-15; F_extinction day*genotype_(3,48)=0.3886, *p=*0.7617; F_extinction day_(2.2,35.5)=0.8638, *p=*0.4403; F_genotype_(1,28)=10.39, *p=*0.0032). **J** AUC analysis revealed increased freezing in DRD^+^ female mice on extinction day 1 with CNO in context A (n=7-14; F_extinction day*genotype_(3,37)=2.568, *p=*0.0691; F_extinction day_(2.2,27.6)=0.2.766, *p=*0.0751; F_genotype_(1,26)=7.944, *p=*0.0091). DRD^+^ mice are KRT14^Cre+^ x hM3Dq^+^, DRD^-^ mice are wildtype littermates. (**p<*0.05, ***p*<0.01, ***p<0.001, *****p<*0.0001).

**Supplemental Video 1**

DRD^+^ mice following CNO (5mg/kg) stimulation of KRT14^cre^ cells.

**Supplemental Video 2**

DRD^-^ mice following CNO (5mg/kg) stimulation of KRT14^cre^ cells.
